# Could China’s journey of malaria elimination extend to Africa?

**DOI:** 10.1186/s40249-022-00978-w

**Published:** 2022-05-16

**Authors:** Duoquan Wang, Shan Lv, Wei Ding, Shenning Lu, Hongwei Zhang, Kokouvi Kassegne, Shang Xia, Lei Duan, Xuejiao Ma, Lulu Huang, Roly Gosling, Joshua Levens, Salim Abdulla, Mutinta Mudenda, Moses Okpeku, Kenneth Kamwi Matengu, Potiandi Serge Diagbouga, Ning Xiao, Xiao-Nong Zhou

**Affiliations:** 1 Chinese Center for Disease Control and Prevention, National Institute of Parasitic Diseases, Shanghai, People’s Republic of China; 2 Chinese Center for Tropical Diseases Research, Shanghai, People’s Republic of China; 3 WHO Collaborating Centre for Tropical Diseases, Shanghai, People’s Republic of China; 4 National Center for International Research on Tropical Diseases, Ministry of Science and Technology, Shanghai, People’s Republic of China; 5 Key Laboratory of Parasite and Vector Biology, Ministry of Health, Shanghai, 200025 People’s Republic of China; 6 School of Global Health, Chinese Center for Tropical Diseases Research, Shanghai Jiao Tong University School of Medicine, Shanghai, China; 7 Department of Parasite Disease Control and Prevention, Henan Province Center for Disease Control and Prevention, Zhengzhou, 450016 People’s Republic of China; 8 Department of Infectious Diseases, Huashan Hospital, State Key Laboratory of Genetic Engineering, Ministry of Education Key Laboratory for Biodiversity Science and Ecological Engineering, Ministry of Education Key Laboratory of Contemporary Anthropology, School of Life Science, Fudan University, Shanghai, 200433 China; 9 Global Health Sciences, Malaria Elimination Initiative, University of California, San Francisco, CA USA; 10 RBM Partnership to End Malaria, Geneva, Switzerland; 11 Ifakara Health Institute, Kiko Avenue, Mikocheni, P. O. Box 78378, Dar es Salaam, Tanzania; 12 National Malaria Elimination Centre, Zambia Ministry of Health, Lusaka, Zambia; 13 Discipline of Genetics, School of Life Sciences, College of Agriculture, Engineering and Sciences, University of KwaZulu-Natal, Durban, South Africa; 14 University of Namibia, Rundu, Namibia; 15 Biomedical Research Laboratory, Institut de Recherche en Sciences de la Santé (IRSS), 03BP7192 Ouagadougou, Burkina Faso

**Keywords:** China, Malaria, Elimination, Efforts, Extend, Africa

## Abstract

World Health Organization (WHO) certified China malaria-free on June 30, 2021, which brightens the goal of global malaria elimination efforts. China contributed its unique innovations to the global community: Artemisinin, discovered by Tu Youyou, has saved millions of lives globally; the “1-3-7” norm developed in 2012, has been adapted in the local contexts of countries in the Southeast Asia and Africa. How to the targets of Global Technical Strategy for Malaria (GTS) 2016−2030. By looking into the malaria control phase, towards elimination phase from 1960 to 2011 in sub-Saharan Africa and China, we found that the gap in malaria burden will widen unless the interventions in Africa are enhanced. It is imperative to identify the key China–Africa cooperation areas on malaria control and elimination, so that synergized efforts could be pooled together to help African countries achieve the elimination goal. The practices from China malaria control and elimination efforts could be leveraged to fast-track malaria elimination efforts in Africa, which makes it possible that the China’s journey of malaria elimination extends to Africa.

**Graphical Abstract:**

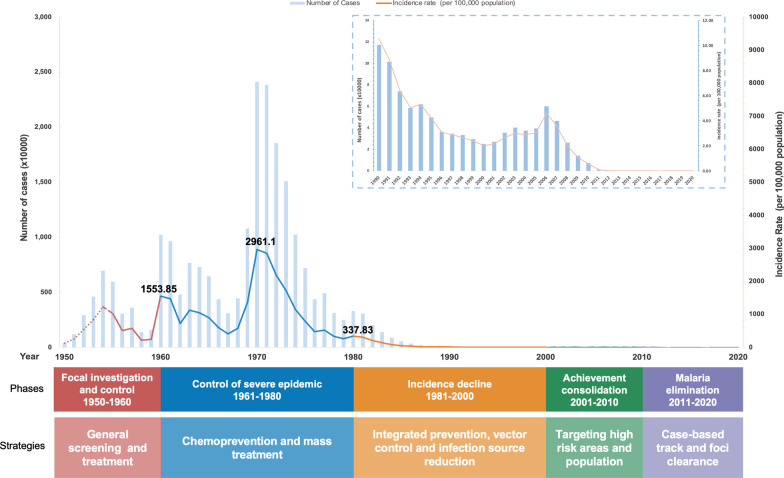

## Background

World Health Organization (WHO) certified China malaria-free on June 30, 2021, which symbolized that the country completed the journey from 30 million indigenous cases annually in the 1940s to zero since 2017 [1]. Undoubtedly, China was of the heaviest malaria burden among the 40 malaria-free countries and territories. But now, it becomes the first malaria-free country in WHO Western Pacific Region [2].

Africa has made significant progress in malaria control during the past two decades. Over 60% of deaths caused by malaria have been reduced in the endemic countries with the critical support from international partners such as the Global Fund to Fight AIDS, Tuberculosis and Malaria, and the USA President’s Malaria Initiative, WHO and the Rolling Back Malaria (RBM) Partnership to End Malaria. However, the progress has been stagnated by interconnected challenges including the operational, technical, and challenges under the impact of coronavirus disease 2019 (COVID-19) pandemic since 2014 and reached a critical juncture with the compounding impact of COVID-19 pandemic. In 2019, an estimated 215 million new malaria infections occurred in Africa, accounting for 94% of the global burden, and the malaria elimination efforts in Africa will accelerate the global malaria elimination goal. However, the targets of Global Technical Strategy for Malaria (GTS) 2016−2030 are under challenge.

The elimination in China brightens the goal of global malaria elimination efforts. During the past decades, China has contributed its unique innovations to the global community: Artemisinin, discovered by Tu Youyou, has saved millions of lives globally; the “1-3-7” norm (whereby case notification within 1 day, case investigation within 3 days and foci investigation and targeted action within 7 days) developed in 2012, has been adapted in the local contexts of countries in Southeast Asia and Africa [1]. Possessed with these innovative tools, China always continues its contribution to Africa malaria elimination efforts. By looking into the malaria control phase towards elimination phase from 1960 to 2011 in sub-Saharan Africa and China, it is shown that the gap in malaria burden will widen without the enhanced interventions in Africa [3]. It is imperative to identify the key China–Africa cooperation areas on malaria control and elimination, so that synergized efforts could be pooled together to help African countries achieve the elimination goal.

### China’s journey of malaria elimination

**Fig. 1:**
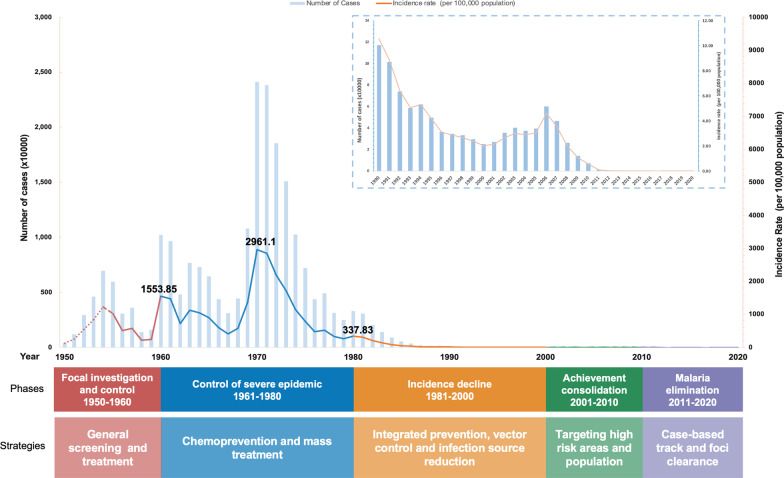
Five phases of China’s malaria control and elimination strategies, 1949−2020

Historically, malaria, one of the five oldest and deadliest human parasitic diseases, left its huge toll on China. Following 70 years of relentless efforts to combat this ancient killer, China has witnessed no single documented case of malaria, originating within its borders since 2017. It is widely recognized that China’s anti-malaria campaigns can be divided into 5 phases (Fig. 1):

- Phase I: Focal investigation and control (1949–1959). With the goal to rapidly reduce malaria mortality and morbidity in the high transmission areas, special mass screening and treatment for the targeted high risk population reduced the national malaria incidence by 57.3% from 505.23/100,000 in 1956 to 215.83/100,000 in 1958.
- Phase II: Control of severe epidemics (1960–1979). The malaria transmission during this phase experienced several serious fluctuations due to the unexpected natural and political episodes, including the two large-scale outbreaks that occurred during the early 1960s and early 1970s. By rolling out the chemoprevention and mass treatment, the malaria incidence was further reduced by 91.3% to 257.54/100,000 in 1979, though the peaking in 1970 over 2961.10/100,000.
- Phase III: Steady decline in incidence (1980–1999). With the continuous reduction in malaria prevalence after 1979, as the severe epidemic was brought under control, the integrated preventive treatment and vector control efforts as well as infection source reduction were adopted afterwards. As a result, the malaria incidence decreased steadily for 20 years and remained below 1/10,000.
- Phase IV: Consolidating previous achievements (2000–2009). Until the end of the 20st century, the malaria was controlled in most areas of China, with cases mostly in the regions which had historically experienced focal endemic outbreaks. Thus, China focused its efforts to target the high-risk areas and populations, especially in the border areas of Yunnan and mountainous areas of Hainan, with the malaria incidence in China brought down to 0.06/10,000 in 2009. This remarkable progress laid an important foundation for China to paradigm shift from malaria control to malaria elimination.
- Phase V: Malaria elimination (2010–now). The National Malaria Elimination Action Plan (NMEAP), officially endorsed in 2010, marked the new beginning of China’s battle against malaria [4]. The national priority of malaria elimination efforts transformed from the community-based interventions to case-based and focus on preventing further potential transmission through the “1-3-7” norm [5] norm.

China’s major experiences that should be adapted by African countries towards malaria control and elimination.

1. Country leadership and whole-of-government approach. Malaria is a notifiable disease in China and it was targeted to be eliminated in the official document in 1956. Since then, malaria control was integrated into China’s national disease control program and subsequently the middle-long term action plans have been implemented up to date. Moreover, China activated the inter-sectoral coordination mechanism with 13 departments of central government to ensure malaria elimination.
2. Mass campaign. The participation of communities in anti-malaria campaign was always a core driver for practice of national strategies and measures. China mobilized and relied on the power of the masses in various regions to carry out the patriotic health campaign, which also created the favorable environment for effective control of mosquito breeding in rice fields, thus greatly reducing the density of local mosquitoes.
3. Tailored interventions. China has taken different combination of interventions in different malaria endemic areas under the principle called “classified guidance”. In the areas that *Anopheles minimus* and *An. anthropophagus* as the main transmission vectors, China adopted the indoor residual spraying once a year or more and the patients were treated and people were provided with preventive chemotherapy simultaneously. In the areas with *An. sinensis* as the main transmission vector, elimination of infectious source was taken as the key intervention, supplemented by vector control.
4. Pilot research before scaling up. Considering the vast territory and population of China, the national malaria control/elimination program launched a series of pilot research and distilled the best practices before scaling up.
5. Adapative management and regional alignment of joint control. Malaria transmission is not restricted by the artificial administrative units. To consolidate the local control efforts, the former Ministry of Health (current National Health Commission) aligned the malaria control interventions across the provinces so that all the parties involved in the joint control activities could facilitate each other, and move forward together under the agreed requirements.
6. Professional resources and grassroots anti-malaria network. The malaria control centers or institutions at different levels and skilled staff composed the anti-malaria system since the mid-1950s. The off-job or in-service health workers and activists at the grassroots level played an important role in health education, patriotic health campaign and malaria control efforts including the blood test, drug dispensary and mosquito eradication.

Although no indigenous case occurs in China, the present achievement is being challenged by continuous imported malaria. From 2017 to 2020, a total of 9287 imported cases were recorded, not only from the border region, but also from the high-burden African countries. In order to consolidate the achievement of malaria elimination, prevent re-establishment and reduce malaria deaths, 13 Ministries of China central government issued the “Administrative Management for the Prevention of Re-establishment of Malaria Transmission” and the Chinese Center for Disease Control and Prevention (China CDC) published the “Technical Program for the Prevention Re-establishment of Malaria Transmission” in home. The strategies and measures for different risk areas have been defined. The new working principle of “government leadership, departmental cooperation, rapid and accurate, joint participation and action“ and the new strategy of “early detection, accurately block transmission” have been developed. To further consolidate the malaria elimination efforts, it is important for China to work with partners globally to mitigate the impact of malaria.

### China–Africa malaria control cooperation practices

**Fig. 2:**
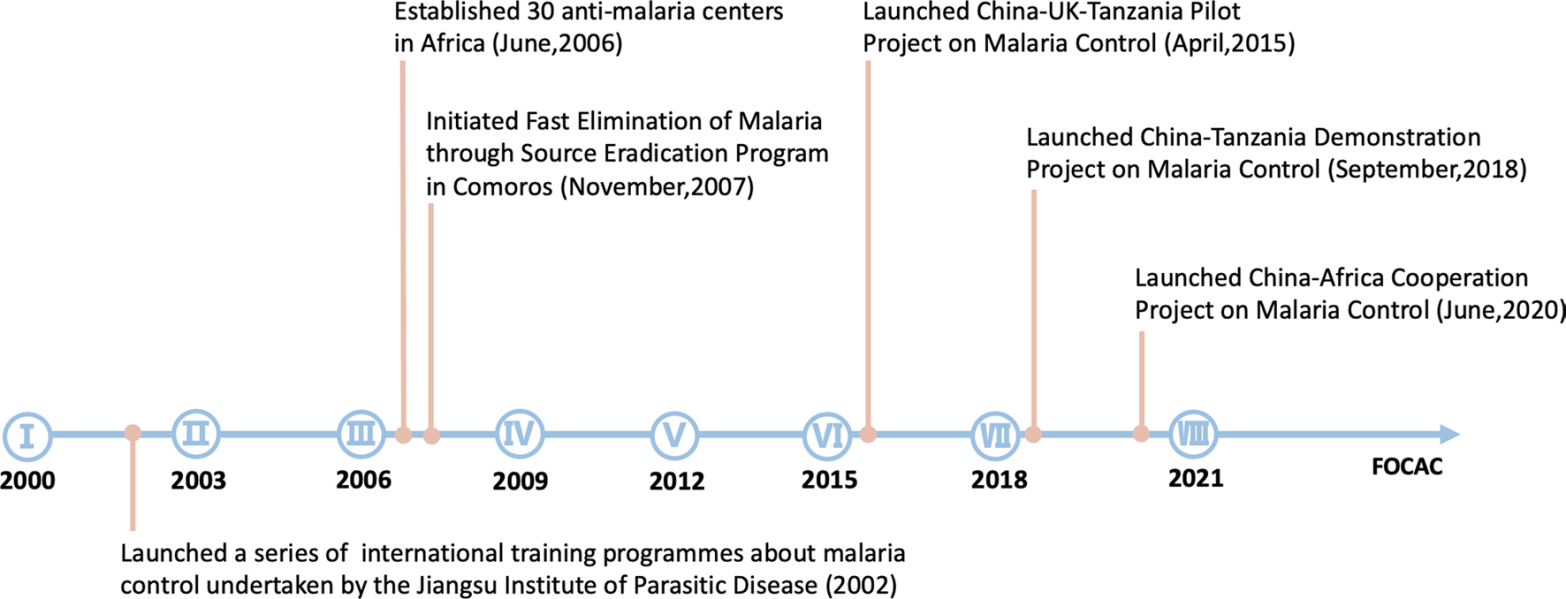
Major China–Africa malaria cooperation projects taken under the FOCAC platform, 2000−2020

The lynchpin of China–Africa malaria cooperation is the Forum on China–Africa Cooperation (FOCAC) initiated at the Ministerial Conference in Beijing in 2000. Since then the China–Africa malaria cooperation has expanded in scale and depth with a series of projects (Fig. 2). The cooperation projects showcase the feasibility, effectiveness, and implications of China’s malaria control and elimination experiences applied in the Africa setting.

The Fast Elimination of Malaria through Source Eradication project in Comoros, a high malaria endemic area, showed remarkable results, which contributed to a 95% reduction in cases of malaria in the country, by launching mass drug administration (MDA) of artemisinin-piperaquine in the local and promoting community engagement. However, the elimination gains was not sustained over the long-term due to lack of continuous surveillance and response in post-elimination era.

The China–UK–Tanzania Pilot Project on Malaria Control (the Pilot Project) between 2015 and 2018 has developed the 1,7-malaria Reactive Community-based Testing and Response (1,7-mRCTR) approach to reduce the malaria burden effectively in the moderate and high transmission areas, which is a locally tailored approach adapted from China’s “1-3-7” norm [5]. Due to the remarkable achievements of the Pilot Project, the China–Tanzania Demonstration Project on Malaria Control (the Demonstration Project), supported by Bill and Melinda Gates Foundation, is now being implemented to validate and scale up 1,7-mRCTR approach.

### Challenges and opportunities for China–Africa cooperation on malaria

Table 1 listed the major challenges of malaria elimination in Africa and the potential cooperation areas with China including implementation constraints, biological challenges and the particular challenges under the impact of COVID-19 pandemic.

**Table 1:** Major challenges of malaria elimination in Africa and potential cooperation areas with China

Major challenges of malaria elimination in Africa	Possible cooperation areas with China
1. Implementation constraints [6] Gaps in the implementation and delivery of malaria interventions and relatively weak health systems, incl. weak organizational and staff capacities, limited resource and staffing Lack of sustainable project-driven interventions and domestic funding Lack of efficient coordination Plateauing funding	Utilize China’s skills in adapting malaria strategies to suit local challenges, such as the use of adaptive forms of 1-3-7 norm, mass drug administration, building specific cadres of community health workers to address missed populations such as in border areas of China Promote inter-sectoral coordination for malaria elimination by sharing China’s experiences Funding opportunities from the Belt and Road Initiative
2. Biological challenges Insecticides resistance and *P. falciparum* resistance to sulfadoxine-pyrimethamine and artemisinin and its derivatives) [7] Increased threat of urban malaria from *Anopheles stephensi* mosquitoes * P. vivax* endemic to Africa has expanded considerably and lack of *P. vivax* diagnosis and treatment tools [8]	Strengthen surveillance on insecticides and anti-malarial resistance by sharing China’s experiences on national malaria reference laboratory system Improve vector surveillance network by introducing and adapting China’s vector control tools Optimize *P. vivax* intervention strategy by introducing and adapting China’s diagnosis and treatment tools
3. COVID-19 co-challenges Maintaining malaria services and antimalarial supply with COVID-19 impact Diagnosis and treatment of malaria and COVID-19 co-infection	Develop the diagnosis and treatment guidelines for malaria and COVID-19 co-infection by sharing Chinese experiences

China–Africa malaria cooperation might face a couple of challenges. First, what has been proved effective from the previous China–Africa initiatives must be adapted into sound malaria intervention policies at the national level in malaria endemic countries. Second, the initiatives should better integrate the project of joint cooperation at both the national and local levels. These cooperation initiatives should strengthen local capacity without imposing an undue burden of new requirements for the national programme. Overall, it is clear from Chinese experience that the whole-of-government approach to malaria elimination pays dividends. Third, the fragmented resources from different ministries and stakeholders in China should be united and well planned for China–Africa malaria control and elimination programmes. These experiences learnt from the previous China–Africa initiatives should help improve the future collaboration.

Despite bottlenecks to be resolved, great potential could be expected from China to help fast track malaria control and elimination course in Africa.

First, regarding the potential cooperation models which are tailored to the needs of African country partners, an integrated and phase-based approach is recommended. This will support country specific collaborations which effectively integrate into the local contexts and align overall trade and development coordination with malaria elimination interventions. China’s support for malaria programmes needs to be embedded into the local existing systems as much as possible, which will provide not only a great deal of flexibility and allow for the necessary oversight, monitoring, reporting, and accountability needed to assure that the Chinese government investments are being effectively deployed and achieve epidemiological impact, but it would also ensure the sustainability of interventions. A designated Chinese technical team on malaria should work with the host-country government coordination mechanisms and the other development partners groups, so as to ensure that investments to end malaria are also aligned with national plans for financing and universal health coverage to promote their long-term sustainability.

Second, the 1,7-mRCTR approach for malaria surveillance and response, which originated from the “1-3-7” norm, a core strategy that led to malaria elimination in China, could be scaled up in African countries. This model embedded with well-designed national reporting system ensures prompt reporting, confirmation, investigation, and response to every malaria case [9]. A local tailored 1,7-mRCTR approach that has been successfully implemented in Southern Tanzania holds a promise for promoting the malaria control and elimination efforts in African settings, which might become the foundation of China–Africa malaria cooperation and reinforce the surveillance and response capacity of African countries.

Third, China’s anti-malaria products mainly the medicines, diagnostics, and vector control tools, meeting the WHO prequalification requirements should shift the marketing to Africa or localize the manufacture in Africa. Donation of medicines, e.g., injectable artesunate for severe malaria and sulphadoxine-pyrimethamine artesunate amodiaquine for the seasonal malaria chemoprevention, may save lives being threatened by malaria infection.

## Conclusions

Africa has made significant progress in malaria control and elimination efforts during the past 20 years together with international efforts. Over 60% deaths caused by malaria has been reduced in the endemic African countries under the support of the international players such as RBM Partnership to End Malaria, the Global Fund to Fight AIDS, Tuberculosis and Malaria, and the USA President’s Malaria Initiative. Since 2014, however, the progress in both cases and death has been stagnated by interconnected challenges. The practices from China malaria control and elimination efforts could be leveraged to fast-track malaria elimination efforts in Africa, which makes it possible that the China’s journey of malaria elimination extends to Africa. China government should concert the resources from different domestic stakeholders and international partners to develop the sustainable anti-malaria programmes aligning with the existing national and local systems in Africa setting.

## Data Availability

Not applicable.

## Abbreviations

WHO: World Health Organization
GTS: Global Technical Strategy for Malaria
RBM: Rolling Back Malaria
NMEAP: National Malaria Elimination Action Plan
China CDC: Chinese Center for Disease Control and Prevention
FOCAC: Forum on China–Africa Cooperation
MDA: Mass drug administration
1,7 mRCTR: 1,7-Malaria Reactive Community-based Testing and Response Approach
